# Severe Wear and multiple Pseudotumor formation due to revision for ceramic head breakage after ceramic-on-ceramic Total hip arthroplasty: a case report

**DOI:** 10.1186/s12891-019-2722-x

**Published:** 2019-07-17

**Authors:** Dan Xing, Chaolei Yang, Rujun Li, Yunfei Hou, Bolong Kou, Hu Li, Jianhao Lin

**Affiliations:** 1 Arthritis Clinic and Research Center, Peking University People’s Hospital, Peking University, Beijing, 100044 China; 2 Arthritis Institute, Peking University, Beijing, China; 3 Orthopedic Department, The First People’s Hospital of Pingdingshan, Henan, China

**Keywords:** Total hip arthroplasty, Revision surgery, Bearing, Pseudotumor, Wear

## Abstract

**Background:**

Head breakage is a serious complication following total hip arthroplasty when using Ceramic on Ceramic bearings surfaces. There is still in controversy about the selection of bearing surfaces when conducting revision surgery.

**Case presentation:**

We describe the case of a fifty-year-old man who had undergone right total hip arthroplasty (THA) with ceramic-on-ceramic prostheses in 2011. After a fall 6 years after the primary procedures, radiographs suggested a ceramic head breakage for revision THA with exchange of metal-on-polyethylene bearing. However, 8 months later, severe metallosis and multiple pseudotumor was confirmed in pelvis and surrounding hip after re-revision THA with ceramic-on-polyethylene prostheses. Analysis of the serum metal ion indicated massive wear of the metal head and erosion of the stem neck and taper.

**Conclusions:**

This case vividly demonstrates metal bearings should be avoided and revision with complete synovectomy and thorough debridement should be performed whenever possible for a fractured ceramic bearing.

## Background

Young age and increased levels of activity have been regarded as independent risk factors for aseptic loosening. Further revision total hip arthroplasty (THA) are required due to metal-on-polyethylene (MoP) or metal-on-metal (MoM) bearings [1]. In recent decades, ceramic-on-ceramic (CoC) bearings have shown superior wear resistance than MoP and MoM bearings. Subsequently, there was a significant shift in usage to ceramic materials due to less wear problems [2–5]. Using CoC bearings has also become increasingly attractive in patients with long life expectancy owing to their biocompatibility properties and greatest wear resistance.

Unfortunately, in using CoC bearings surfaces, ceramic head breakage is a serious complication following THA [6–8]. Furthermore, the revision THA can prove very challenging, because the ceramic particles are embedded into the surrounding tissues which subsequently results in rapid implant wear or failure [7, 9]. Although several studies recommended some interventions for revision THA, including limited patient’s physical activity, early surgery, removal of ceramic particles, complete synovectomy and replace or retain the well-fixed implants [7, 8, 10], there was still in controversy about the selection of bearing surfaces and femoral stem retention [11].

Here, we describe the case of a fifty-year-old man who had undergone right THA with CoC prostheses (Smith&nephew, Switzerland) in 2011. Six years later, he presented at our clinic with crunching noise after a fall on the right hip. Radiographic examination suggested a fracture of ceramic head of the right THA. Revision THA was conducted with MoP bearings prostheses (Smith&nephew, Switzerland) in 2017, but without revising the stem. Eight months later, he was admitted to our hospital only with low fever. Catastrophic metallosis and non-infectious pseudotumor was confirmed after re-revision THA with CoP prostheses (Smith&nephew, Switzerland) in 2018.

The patient was informed that data concerning the case would be submitted for publication, and he provided written consent.

### Case report

**Fig. 1:**
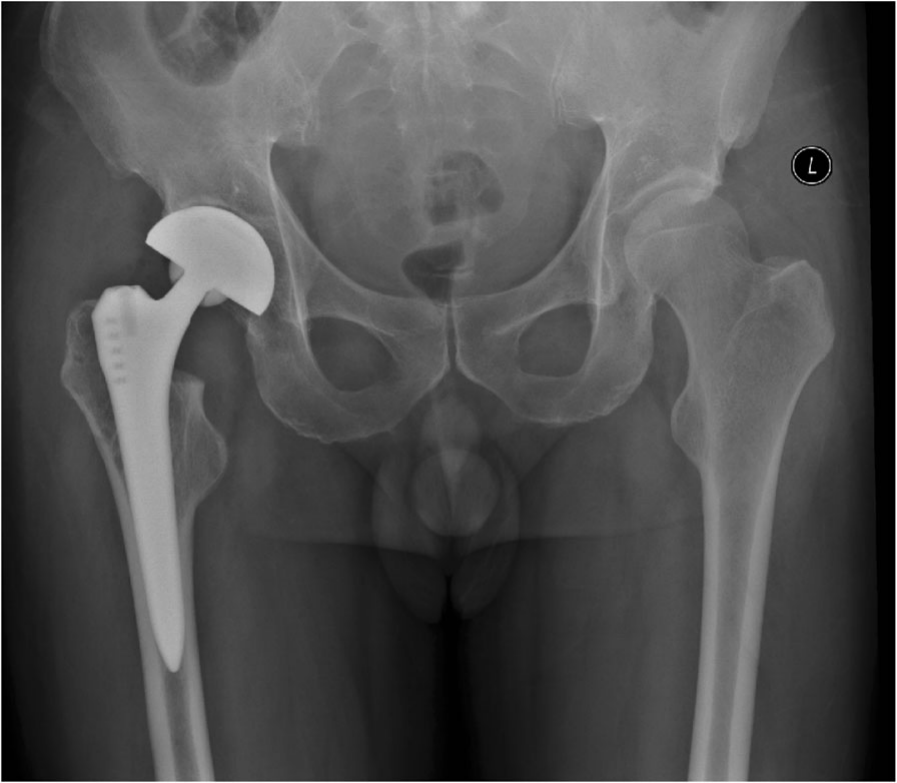
Anteroposterior radiograph of right cementless CoC total hip replacements

In March 2011, a fifty-year-old man underwent a THA with CoC bearings (BIOLOX, Smith & nephew, Switzerland) on the right side at our clinic because of femoral head necrosis after internal fixation for femoral neck fracture for 4 years. In this surgery, a titanium alloy size-5Standard Stem (Smith & nephew) was used, along with a 28-mm fourth generation ceramic head (Smith & nephew). The acetabular side consisted of atitanium alloy Shell (Smith & nephew) with a ceramic liner that had a ceramic bearing interface (Fig. 1).

**Fig. 2:**
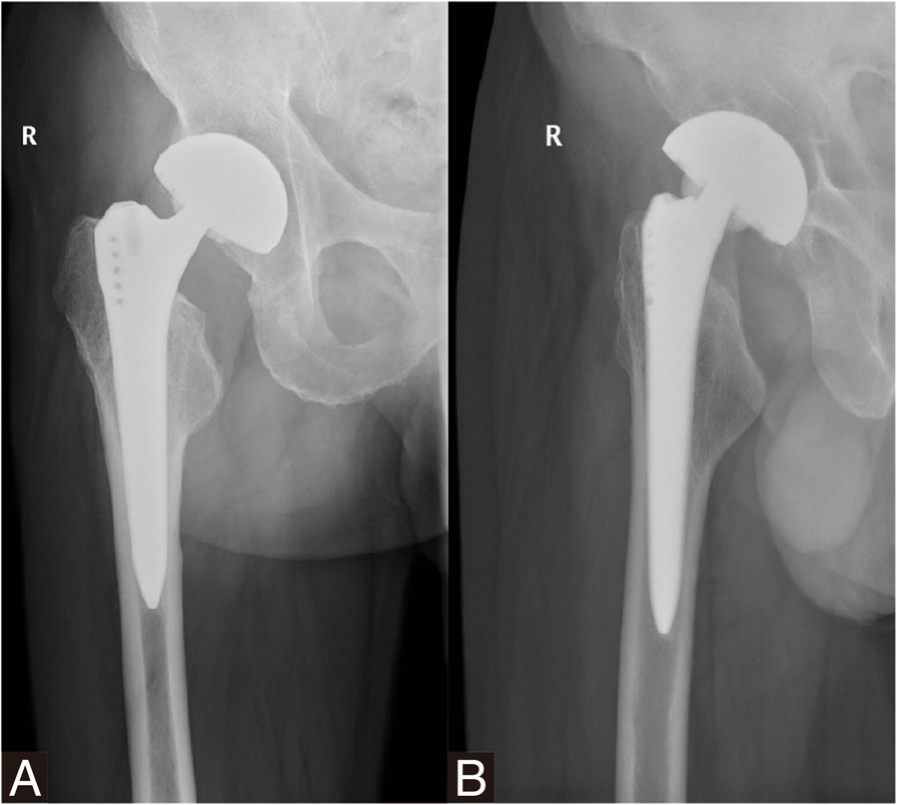
Radiograph showing fracture of the ceramic femoral head with marked fragmentation (**a**) Anteroposterior view. **b** Lateral view

In September 2017, he presented at our clinic after an accidental fall on the right hip complaining of crunching noise but without pain, swelling, and disability. Examination of right hip showed normal range of motion. There was no pain during range of motion testing of right hip. He was diagnosed with ceramic head breakage by radiographic examination (Fig. 2). Despite the surgeon’s strong recommendation based on the fracture of the ceramic head, the patient refused revision at that time.

**Fig. 3:**
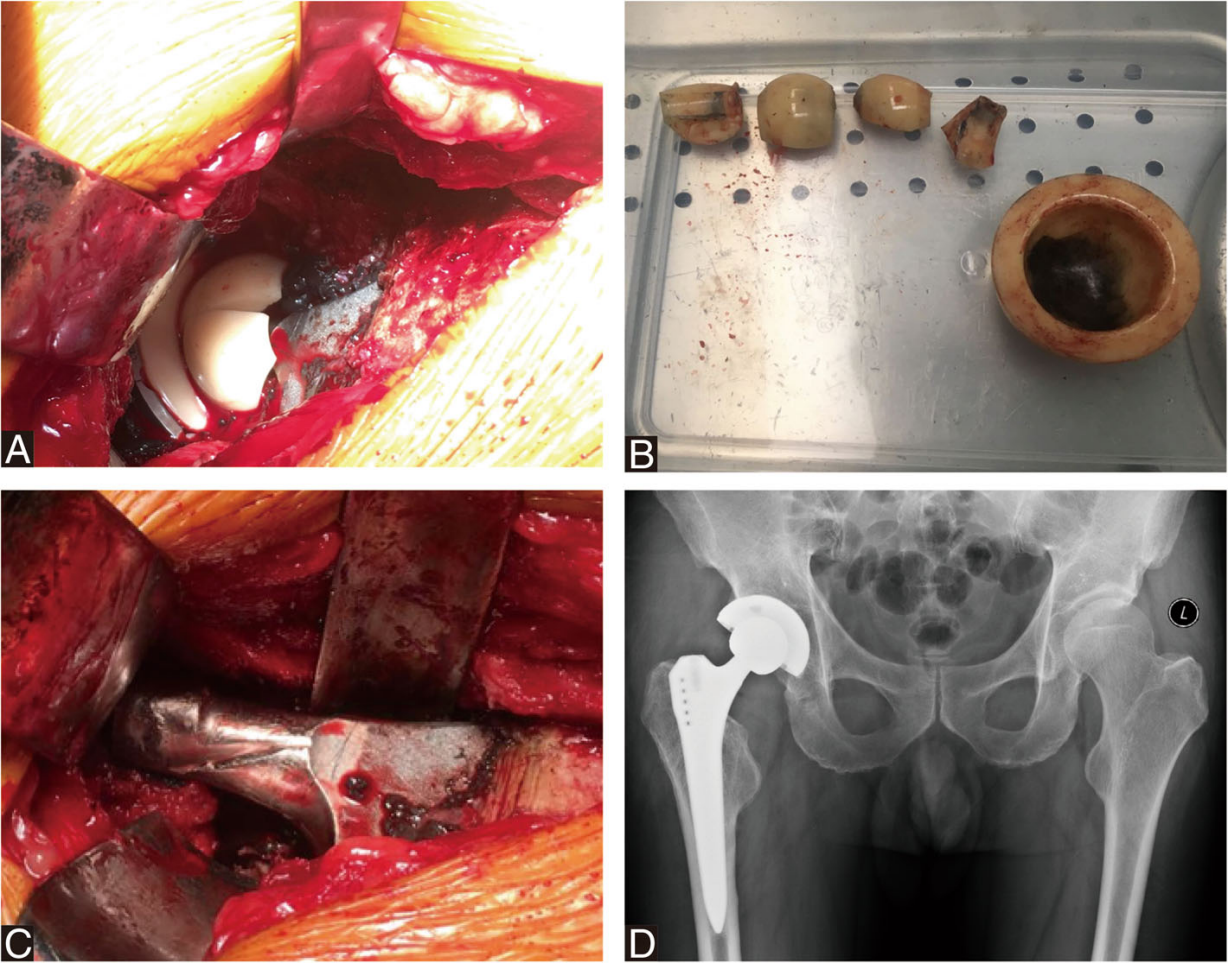
Revision MoP total hip arthroplasty for ceramic head breakage. **a**-**b** Intraoperative photograph demonstrating ceramic head breakage and (**c**) severe wear of the taper and neck of femoral stem. **d** Postoperative anteroposterior radiograph after revision with metal head and polyethylene liner

Because of continuous crepitus in the right hip, a revision of the right THA was performed in November 2017. During revision, intraoperative findings included wear of the taper and neck of stem, a multifragmented head and intact ceramic liner. Considering the satisfied stability of stem and acetabular component, we performed revision of the head and liner with MoP with retaining the well-fixed stem and acetabular component (Fig. 3). After aggressive debridement and thorough lavage, the incision was closed.

After 8 months, he was admitted to two hospitals consecutively complaining of low fever, loss of appetite and weight loss. Clinical examination revealed painless normal range of motion of the bilateral hips. No signs of infection were observed. X-ray showed huge soft tissue mass around right hip prosthesis. Computed tomographic (CT) scan at admission showed a mass extending to the pelvic without bone destruction. Laboratory analysis of inflammatory markers showed slightly elevated white cell count at 10.42 × 10^9^/L (N: 87.6%), C-reactive protein at 46.92 mg/L, and erythrocyte sedimentation rate at 7 mm/h. Blood test for malignant tumors markers showed normal indicator. Based on his long history of THA and radiographic findings, clinical diagnosis of implants-associated chronic inflammatory mass was made. Thus, the patient was admitted to our clinic for further treatments.

**Fig. 4:**
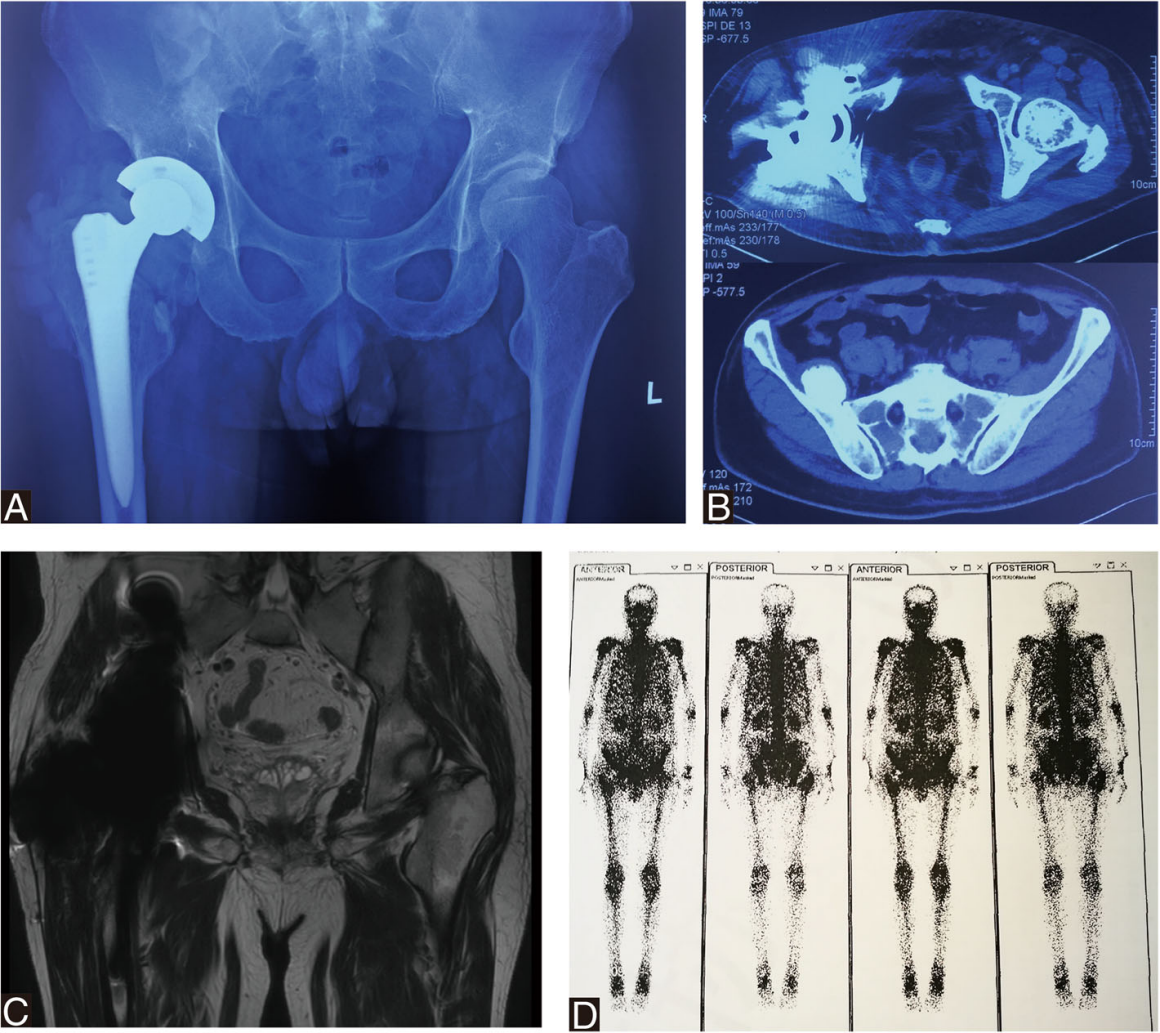
Pseudotumor formation after revision MoP Total hip arthroplasty. **a** Anteroposterior radiograph showing extensive soft tissue mass around the right hip prosthesis and a mass in pelvis. **b** A transaxial view of the CT showed extensive right hip high density shadow and a high density mass in pelvis. **c** MRI T2image showed significant artifact in the region of the right hip MoM prosthesis. Surrounding the prosthesis was extensive soft tissue and muscle edema affecting the gluteus muscles, piriformis, rectus femoris, obturator externus, pectineus, and the adductor bundle. **d** ^99m^Tc-MDP bone scintigraphy showed a photopenic area in the region of the right hip prosthesis that appears to extend into the surrounding soft tissues

X-ray and CT scan confirmed extensive muscle and soft tissue mass around the right hip prosthesis and in pelvis. Coronal T2magnetic resonance image showed significant artifact in the region of right hip joint and in the pelvis abutting the medial wall of the right acetabulum. Surrounding the prosthesis was extensive soft tissue and muscle edema affecting the adductor bundle, gluteus muscles, rectus femoris muscle and obturator externus. 99mTc-MDP bone scan indicated benign tumor without distant metastases in pelvis (Fig. 4). A provisional diagnosis of necrotic pseudotumor was suggested. The patient’s serum C-reactive protein was 33.42 mg/L and erythrocyte sedimentation rate was 9 mm/h. His complete white cell count was elevated at 14.10 × 10^9^/L (N: 81.0%). Serum ion analysis was conducted by inductively coupled plasma mass spectrometry (ICP-MS, ThermoFisher, USA). Serum ion concentration was shown in Table 1. Serum cobalt, chromium, titanium levels were significantly elevated 5 days preoperatively (serum Co = 1065.39 μ g/L, serum Cr = 19.58 μg/L, serum Ti = 106.27 μg/L). Given the clinical and imaging findings and serum analysis, combined with a history of CoC to MoP revision THA, we deemed that this patient’s symptoms were likely due to severe metallos is caused by third particle wear when a fractured ceramic head and subsequent multiple pseudotumor formation.

**Table 1:** Metal ion concentrations in the serum or in the periprosthetic tissue

Tissue (time point)	Cobalt (ng/ml)	Chromium (ng/ml)	Molybdenum (ng/ml)	Titanium (ng/ml)
Serum (5 days pre-operation)	1065.39	19.58	3.21	106.27
Surrounding tissue (intraoperation)	148.01	766.20	39.80	10.05
Dark gray fluid (intraoperation)	21389.71	89212.02	5431.69	279.83
Serum (2 months post-operation)	453.07	9.52	1.23	150.20

Norm values: Cobalt: < 2.5; Chromium < 2.03; Molybdenum 0.5–2; Titanium 2–10 (ng/ml)

**Fig. 5:**
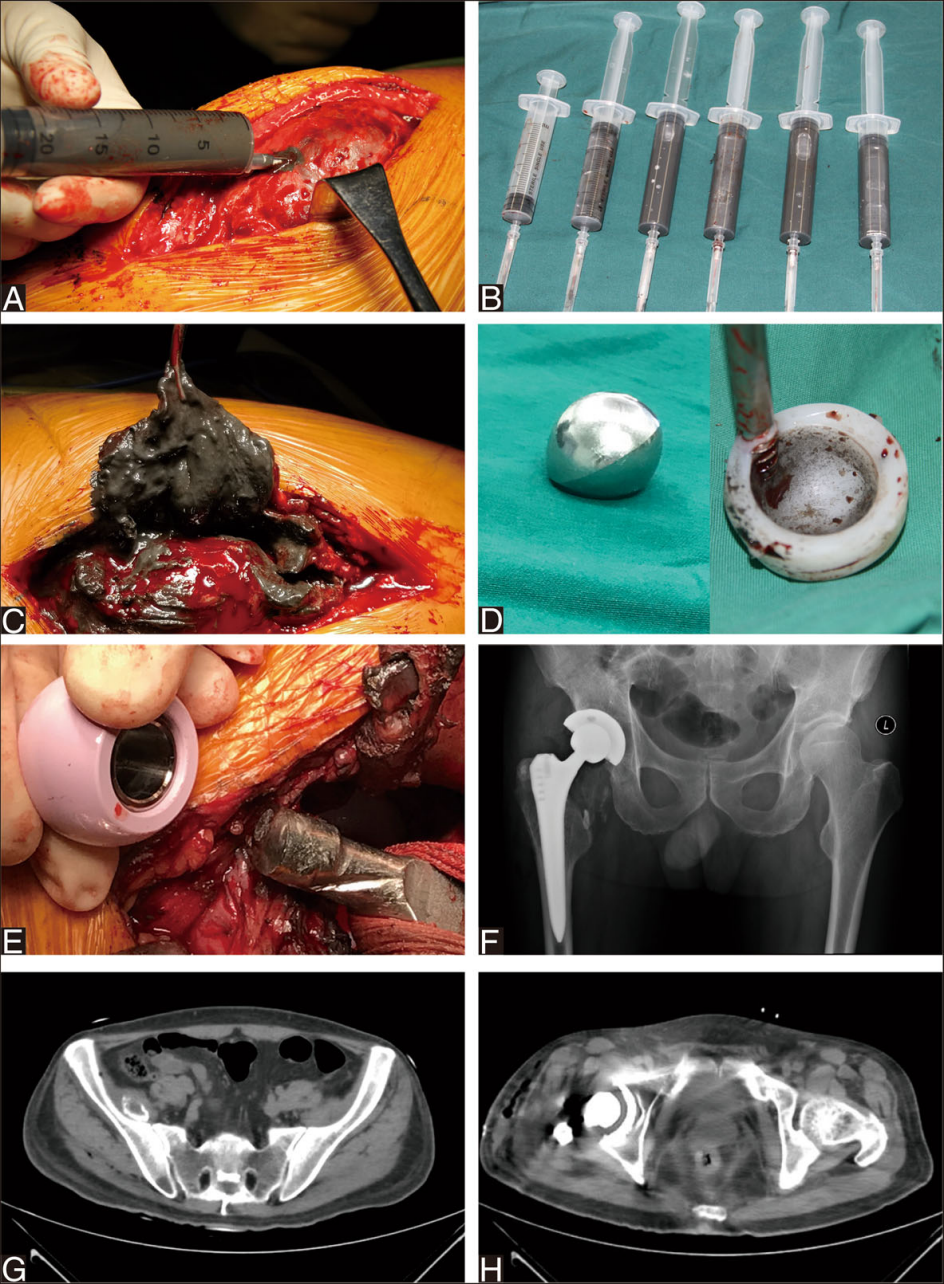
Re-revision CoP total hip arthroplasty for pseudotumor. **a**-**b** Dark gray drainage from surrounding synovial sacs was about 100 ml. **c** Dark gray tissue was visible around the right hip joint. **d** Photograph demonstrating metal head and polyethylene liner with severe wear. **e** A ceramic head along with a titanium sleeve was placed on the previous femoral stem. **f**-**h** Anteroposterior radiograph and transaxial view of the CT at immediate postoperatively showed unremarkable pseudotumor surrounding hip and a cystic wall in pelvis

**Fig. 6:**
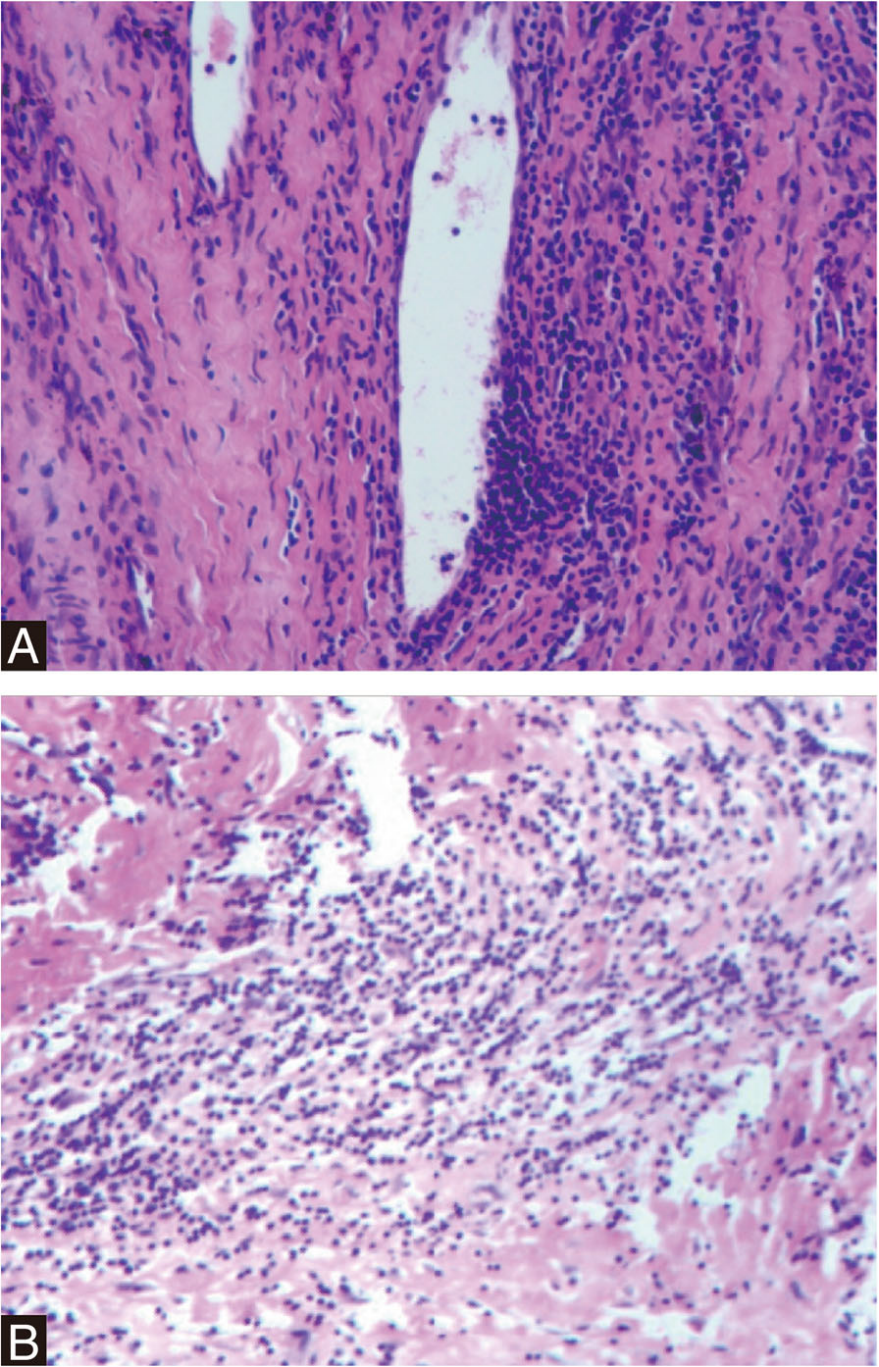
Postoperative histopathology. **a** Perivascular lymphocytes indicated inflammatory response to metal particles. **b** Histopathology showed necrotic tissue with fibrosis, fibrin material and a few scattered lymphocytes

Surgical exploration with re-revision THA for severe wear and multiple pseudotumor formation was discussed with the patient and he underwent a re-revision THA by a lateral approach. During re-revision, extensive metallos is and dark gray tissue was visible around the joint, suggestive of multiple pseudotumor. The total volume of dark gray drainage from surrounding synovial sacs was about 100 ml. The pseudotumors and surrounding synovial sacs around prosthesis were then excised. The cystic soft tissue swelling herniated out under the inguinal ligament into the right pelvis. The dark gray fluid from pelvis was debrided aggressively and lavaged thoroughly. The intraoperative frozen section of the excised mass revealed less than 5 polymorphonuclear leukocytes per high-power field in all the three specimens. The patient’s hip was dislocated. The acetabular shell and femoral stem were well fixed, and therefore, these components were retained. A severe wear was noted around the metal head and polyethylene liner (Fig. 5). The taper exhibited wear without fracture. Thus, on the femoral side, a ceramic head (32/+ 3.5 mm) (Zimmer, Switzerland) was used, along with a titanium sleeve (Zimmer, Switzerland). On the acetabular side, the polyethylene liner (Smith & nephew, Switzerland) was exchanged. The components were verified as being stable and synovectomy and extensive debridement were conducted before closing the wound. For excised mass, the histopathology analysis supported inflammatory response to metal particles, indicated by the presence of perivascular lymphocytes and necrotic tissue with fibrosis, fibrin material (Fig. 6).

**Fig. 7:**
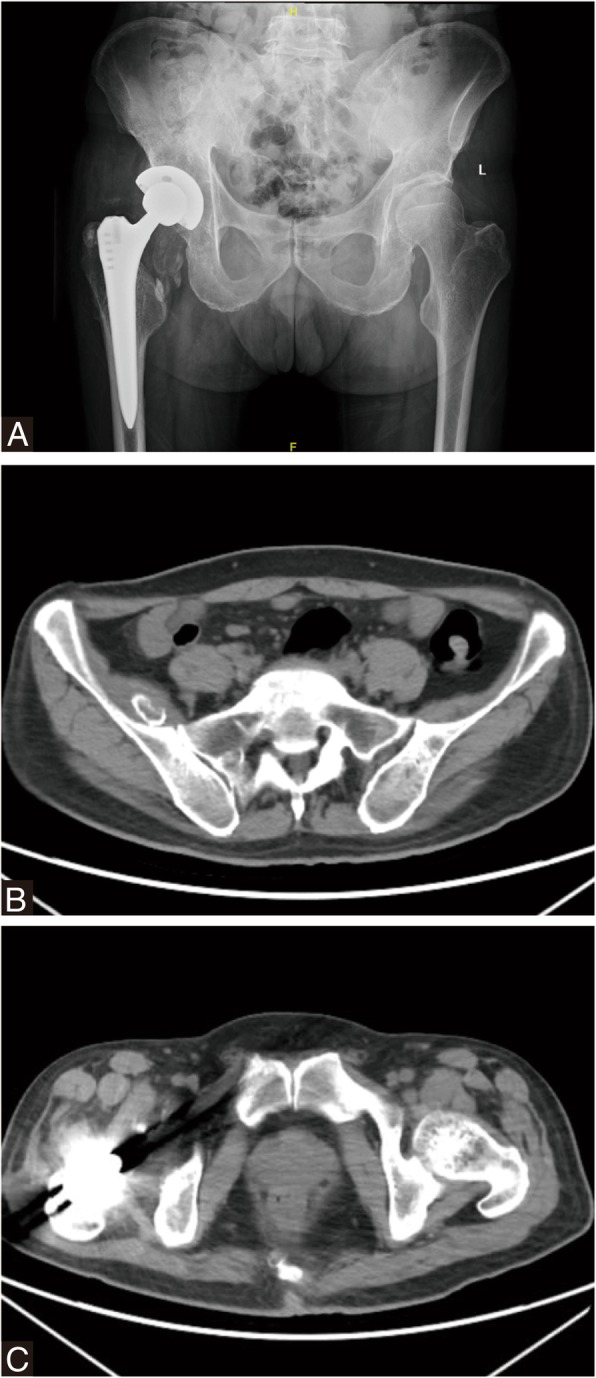
Two months following-up after re-revision surgery. **a** Radiograph showing unremarkable pseudotumor surrounding hip and mass in pelvis. **b**-**c** A transaxial view of the CT showed a capsule wall in pelvis and lower density shadow surrounding the right hip

Intraoperative metalion of periprosthetic tissue and dark gray fluid analysis was done, showing increased cobalt, chromium, molybdenum and titanium concentration (Table 1). It indicated metal head and taper erosion and consequent metal toxicity. At 2 months, a repeat serum metal ion analysis revealed a decrease in Co and Cr ions levels with their values being 453.07 and 9.52 μg/L, respectively (Table 1). X-ray at immediate and 2 months postoperatively were unremarkable, while CT scan still showed cystic wall compares the solid pseudotumor preoperatively in pelvis (Fig. 7).

## Discussion and conclusions

The pseudotumor formation in the present case was caused by third particle wear on metal head after a fractured ceramic head breakage, which was indicated by increased metal ion release of cobalt and chromium. Furthermore, the increased amount of titanium was confirmed by serum analysis. The metal ion release of titanium may be originated from tribocorrosion of the stem neck and taper caused by cuts from sharper ceramic [12]. To the best of our knowledge, the present case is the first to describe rapid multi-pseudotumors formation and large pelvic mass due to severe wear after a revision MoP THA managed with re-revision arthroplasty.

Although CoC bearings are associated with low wear rates, breakage of head is still a concern. Breakage of ceramic head may associated with some intra- or post-operative factors, including excess hoop stress from impaction, component malposition, increased cup inclination, taper design, taper mismatch and impingement [13]. The use of 28-mm head and shorter neck during surgery are other potential risk factors for head breakage [9]. Lee et al. [14] reported that large-diameter heads were associated with a lower rate of fracture compared to smaller-diameter femoral heads in nearly 6 million hip implants. Moreover, revision THA conducted due to fractured ceramic implants is still to be complicated [11]. The main puzzle is that complete removal of the ceramic debris during revision is often impossible. Owing to no consensus on choice of implantation, metal head was used in the first revision in the present case. As demonstrated in the present study, the metal head was scraped by third particle from fractured ceramic head breakage. Therefore, wear of the revised articulation by retained ceramic debris is often catastrophic. As showed in the present case, a ceramic head with 28 mm diameter ceramic head was used in primary surgery which may increase the risk of breakage. Furthermore, the presentation of symptoms occurred only 8 months after the revision MoP THA. The duration was shorter than that of average soft tissue reactions involving a MoP bearing (range from 3 to 26 years) [15]. The severity of wear is attributed to the following factors, patient’s physical activity, kept non-weight bearing, postponed revision THA after ceramic breakage, complete synovectomy, thorough lavage and choice of bearing surface. Although complete synovectomy and thorough lavage were conducted in revision arthroplasty in this case, small ceramic debris cannot be removed completely.

Currently, MoP or CoP/CoC bearings are all used in revision THA due to fracture of implants. Although there is no guideline on the best bearing for revision THA following fracture of the ceramic head, CoP or CoC bearings seem to reduce the risk of third-body wear [8]. Thus, as suggested by several studies, MoP bearing should be avoided because of the risk of head erosion and pseudotumor formation [8, 11, 16]. The present case clearly demonstrates that third-body wear and correspondingly increased release of metal ions (cobalt and chromium) are caused by MoP bearing in revision THA after ceramic head fracture. Thus, pseudotumor was formed rapidly in periprosthetic tissue and in pelvic.

In this case, the femoral stem was retained in revision THA and in re-revision surgery after intraoperative inspection of the stability of the stem. However, titanium alloy sleeve was not used in revision MoP THA, which could lead to further erosion of the taper by unevenness. It was indicated by increased metal ion concentrations of titanium. In treating pseudotumour during re-revision, non-metal bearing is required [17], thus, the patient was revised with CoP with a titanium alloy sleeve. It is our preference to use a titanium alloy sleeve when a ceramic head is being placed on a previously used femoral stem taper, because sleeve can create a smooth taper surface and prevent repeat fracture of the revised head. In spite of using titanium alloy sleeve in re-revision THA, whether or not revise the femoral stem with scratches or corrosion on neck and taper was still unclear [8, 18]. Thus, femoral stem was always retained in this case. Furthermore, since a titanium alloy stem with wear of the taper and neck was still present in crevice environment and erosion, the patient is still needed for serum metal ion analysis during follow-up, especially the concentration of titanium.

Diagnosing hip joint infection remains a major challenge as there is no test with absolute accuracy [19]. The diagnosis is commonly based on a combination of clinical findings, laboratory test, microbiological culture, histological evaluation, and intraoperative findings. Based on the preoperative test and intraoperative findings, the current case did not meet the new evidence-based criteria for diagnosing infection after joint arthroplasty [20]. Thus, we ruled out the possibility of infection in differential diagnosis.

The case described herein clearly demonstrates that MoP bearings in revision THA for ceramic head breakage can cause severe wear and increased release of metal ions with correspondingly multiple pseudotumor formation. Early immobilization and revision surgery as soon as possible are strongly recommended after breakage of ceramic head to prevent further formation of ceramic debris. Even small amounts of remaining debris with highest hardness could be deleterious for the outcomes after revision THA with metal components. Thus, revision to CoC or CoP bearing with complete synovectomy and thorough debridement of the fractured ceramic fragments should be performed. The well-fixed femoral stem could be retained when the damage to the neck and taper is not beyond simple scratches or corrosion. A titanium alloy sleeve should be placed on the retained femoral stem taper. The monitor for metal ions concentration in serum is still required during follow-up.

## Data Availability

The final dataset will be available from the corresponding author.

## Abbreviations

CoC: Ceramic-on-ceramic
MoM: metal-on-metal
MoP: metal-on-polyethylene
THA: total hip arthroplasty
